# Speciation Reversal in European Whitefish (*Coregonus lavaretus* (L.)) Caused by Competitor Invasion

**DOI:** 10.1371/journal.pone.0091208

**Published:** 2014-03-13

**Authors:** Shripathi Bhat, Per-Arne Amundsen, Rune Knudsen, Karl Øystein Gjelland, Svein-Erik Fevolden, Louis Bernatchez, Kim Præbel

**Affiliations:** 1 Department of Arctic and Marine Biology, University of Tromsø, Tromsø, Norway; 2 Institut de Biologie Intégrative et des Systèmes (IBIS), Université Laval, Québec, Canada; 3 Norwegian Institute for Nature Research, Tromsø, Norway; The University of Queensland, St. Lucia, Australia

## Abstract

Invasion of exotic species has caused the loss of biodiversity and imparts evolutionary and ecological changes in the introduced systems. In northern Fennoscandia, European whitefish (*Coregonus lavaretus* (L.)) is a highly polymorphic species displaying adaptive radiations into partially reproductively isolated and thus genetically differentiated sympatric morphs utilizing the planktivorous and benthivorous food niche in many lakes. In 1993, Lake Skrukkebukta was invaded by vendace (*Coregonus albula* (L.)) which is a zooplanktivorous specialist. The vendace displaced the densely rakered whitefish from its preferred pelagic niche to the benthic habitat harbouring the large sparsely rakered whitefish. In this study, we investigate the potential influence of the vendace invasion on the breakdown of reproductive isolation between the two whitefish morphs. We inferred the genotypic and phenotypic differentiation between the two morphs collected at the arrival (1993) and 15 years after (2008) the vendace invasion using 16 microsatellite loci and gill raker numbers, the most distinctive adaptive phenotypic trait between them. The comparison of gill raker number distributions revealed two modes growing closer over 15 years following the invasion. Bayesian analyses of genotypes revealed that the two genetically distinct whitefish morphs that existed in 1993 had collapsed into a single population in 2008. The decline in association between the gill raker numbers and admixture values over 15 years corroborates the findings from the Bayesian analysis. Our study thus suggests an apparent decrease of reproductive isolation in a morph-pair of European whitefish within 15 years (≃ 3 generations) following the invasion of a superior trophic competitor (vendace) in a subarctic lake, reflecting a situation of “speciation in reverse”.

## Introduction

Reproductive isolation is a process that impedes the exchange of genes between the members of distinct populations [1]. In ecological speciation, reproductive isolation evolves as a result of adaptive divergence between different sub-populations in response to divergent natural selection within a population [1], [2]. An important promoter of divergent natural selection in ecological speciation is the availability of various ecological opportunities. This provides alternative ecological niches that may favour different behavioral and morphological adaptations which eventually may lead to genetic divergence and reproductive isolation of different morph types [3]–[5]. The reproductive isolation may include both pre-zygotic and post-zygotic isolation mechanisms (Table 1). Pre-zygotic isolation mechanisms mainly involve spatial and temporal isolation (e.g. different spawning sites and time) and sexual selection [6]. The temporal and spatial pre-zygotic isolation mechanism may be important as it restricts gene flow among populations [7]. Sexual selection also represents a potential driver of reproductive isolation (mate choice) [6], [8]–[11]. In fish, premating reproductive isolation mechanisms are usually weak and may permit occasional gene flow among populations [12], [13]. However, the strength of pre-zygotic reproductive isolation mechanisms depends heavily on the environmental and ecological conditions which the isolation barriers are built upon [14]. For example in cichlids and Alpine whitefish, eutrophication has changed the reproductive strategies of species [11], [15], whereas the American signal crayfish has altered the reproductive behavior of males of three-spined sticklebacks [16], resulting in “speciation reversal” [17]. Post-zygotic isolation mechanisms may include extrinsic and intrinsic post-zygotic mechansims. Extrinsic mechansims may involve ecological inviability, whereas intrinsic mechanisms include hybrid inviability and hybrid sterility [1]. Such mechanisms have been documented in three-spined sticklebacks (*Gasterosteus aculeatus*), lake whitefish (*Coregonus clupeaformis*), and Alpine whitefish (*Coregonus* spp.) [18]–[20].

**Table 1:** Putative reproductive isolation mechansims in European whitefish (*Coregonus lavaretus*) and other post-glacial fish.

Classification of putative reproductiveisolation mechanisms	Components	References
**Pre-zygotic isolation mechansims**		
1. Temporal isolation	Different spawning time	[22], [37]
2. Habitat isolation	Different spawning ground or microhabitat segregation	[17], [22], [44], [45]
3. Mate choice	Size based assortative mating	[46]–[48]
**Post-zygotic isolation mechansims**		
*Extrinsic*		
Ecological inviability and incompatibilities	Lack of ecological niches for intermediates, reduced feeding efficiency dueto non-optimal feeding apparatus, asynchronous hatching time, emergence	[19], [20]
*Intrinsic*		
Hybrid inviability	Lethal or partial developmental abnormalities or embryonic mortalitiesin hybrid offsprings due to the genomic incompatibilities	[18], [49]–[51]

Sympatric morph-pairs with partial reproductive isolation frequently occur among post-glacial freshwater fish species such as whitefish *Coregonus* spp. and Arctic charr *Salvelinus alpinus* (L.) [18], [21], [22]. Due to the young geological age, these systems are considered to constitute an early phase of the speciation process [22], [23]. However, anthropogenic impacts like climate change, habitat destruction, and introductions of non-native species may cause instability of native ecological conditions that the pre-mating isolation mechanisms between morphs or species-pairs are dependent upon [15], [17], [24]–[27]. This may result in breakdown of weak pre-zygotic reproductive isolation, inducing hybridization between closely related species or morph-pairs [4], [17]. Increased hybridization between sympatric species following environmental disturbances has been reported in both plants and animals [26], [28], [29].

In northern Fennoscandian lakes, a morph-pair of whitefish referred to as the densely–rakered (DR) and large sparsely–rakered (LSR) whitefish, commonly occur in sympatry [30]–[32]. The DR whitefish is characterised by numerous long, thin and densely packed gill rakers, whereas the LSR whitefish has fewer, shorter, and more sparsely packed gill rakers and usually a larger body size. The DR whitefish generally occupies the pelagic zone feeding predominantly on zooplankton, whereas the LSR whitefish predominantly feeds on zoobenthos in littoral habitats [30]. This use of divergent niches has repeatedly been observed to promote phenotypic and partial reproductive isolation, thus resulting in genotypic differentiation between the two sympatric whitefish morphs across lakes in northern Fennoscandia [21], [32]–[34].

Vendace *Coregonus albula* (L.) is a pelagic, highly specialized zooplankton feeder known to be competitively superior to whitefish in zooplankton foraging [35]–[38]. Vendace were intentionally and repeatedly translocated into the tributaries of Lake Inari in northern Finland in 1964–66 [39], [40] and by the 1980’s, a large population of vendace was established in the lake. From Lake Inari, vendace migrated downstream into the Pasvik watercourse where the species was observed for the first time in 1989 [38]. In 1993, a few vendace were recorded in Lake Skrukkebukta in the lower part of the watercourse [38]. Since then, the vendace population in L. Skrukkebukta has been steadily growing [41]. Long-term studies of fish and zooplankton following the vendace invasion have demonstrated a strong impact of the invader on the size-distribution and abundance of zooplankton [42], which in turn have resulted in a diet shift of the DR whitefish from zooplankton to zoobenthos combined with a competitive relegation from the pelagic to the littoral habitat [35], [38], [43].

Despite the fact that sympatric DR and LSR whitefish are partially reproductively isolated and genetically differentiated [21], [34], the competitive displacement of the DR whitefish from the pelagic into the littoral habitat, which is inhabited by the LSR whitefish, may have enhanced the probability of weakening pre-zygotic isolation between DR and LSR whitefish. Moreover, the overall abundance of the DR whitefish has in some lakes been reduced to around 10% of its pre-invasion value [35]. This raises the hypothesis that the vendace invasion in the watercourse may have triggered breakdown of reproductive isolation between the DR and LSR whitefish. Here, we aimed at testing this hypothesis by contrasting multilocus genotype data from the DR and LSR whitefish in Lake Skrukkebukta at the arrival (1993) and 15 years after (2008) the invasion of vendace. We predicted that while the DR and LSR whitefish would be phenotypically and genetically differentiated prior to the vendace invasion, a breakdown of reproductive isolation and species collapse may have occurred following the invasion (but see [15], [17]).

## Methods

### Study Area and Sample Collection

**Figure 1:**
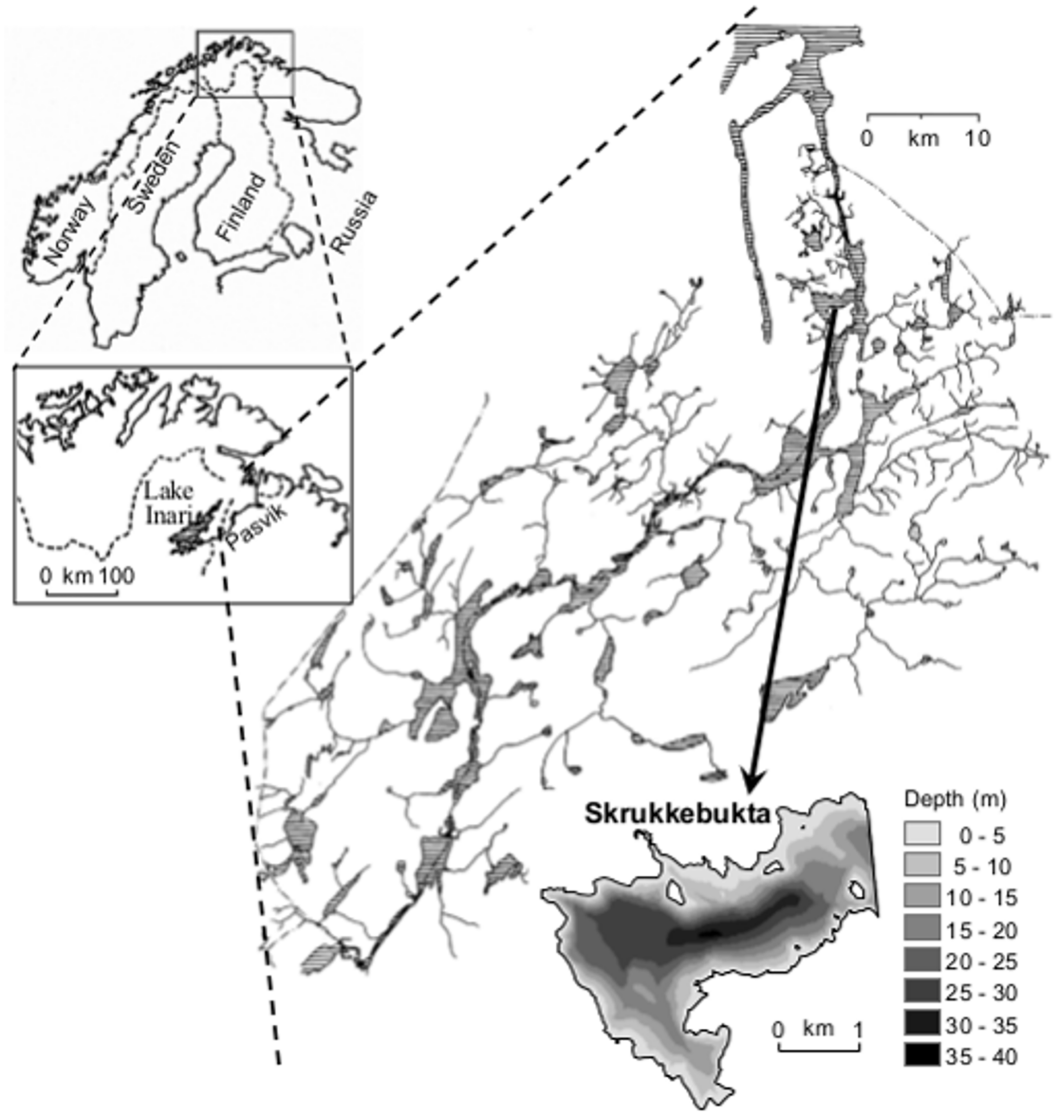
Map of Pasvik watercourse, Finnmark county, northern Norway. Black arrow indicates the location of Lake Skrukkebukta in Pasvik water course.

This study was carried out in the dimictic and oligotrophic Lake Skrukkebukta situated in the Pasvik watercourse, Finnmark county, northern Norway (Figure 1). Lake Skrukkebukta (69° 33′ N, 30°7′ E; 21 m a.s.l.) has an area of 6.6 km ^2^, and a maximum depth of 37 m. In addition to whitefish, the lake harbours perch (*Perca fluviatilis* L.), pike (*Esox lucius* L.), brown trout (*Salmo trutta* L.), burbot (*Lota lota* (L.)) and nine-spined stickleback (*Pungitius pungitius* (L.)). The dataset used for this study consists of gill samples from two sampling years; at the arrival of vendace (1993) and after its invasion and establishment as a superior pelagic feeder (2008). Fish were sampled in the pelagic, littoral and profundal zones using 40 m long gill nets with eight sections of 5 m each, with different mesh sizes of 10, 12.5, 15, 18.5, 22, 26, 35 and 45 mm (knot to knot). In the pelagic zone, 6 m deep floating nets were used, whereas in the littoral and profundal zones, 1.5 m deep bottom nets were employed. The whitefish were classified in the field into either DR or LSR whitefish based on the overall gill raker and body morphology [30]–[32], [38]. In a study using a long term dataset, Siwertsson *et al* [52] revealed that the gill raker numbers is a stable phenotypical trait in European whitefish (*Coregonus lavaretus*) populations in subarctic lakes and constitutes a reliable means to classify morphs of European whitefish. The gill arches were removed and preserved in 96% ethanol for further analysis. The gill rakers on the first left branchial arch were counted under dissection microscope. For the genetic analyses we used 93 fish from year 1993 and 96 fish from year 2008.

Fish were euthanized by means of cerebral concussion prior to sample collection. A fishing permission is required from the fishing right owner, which on Government land in Finnmark county is the County Governor of Finnmark with legal authority through LOV 1992-05-15 nr 47, §13. Accordingly, we obtained permissions for the gill net fishing in Lake Skrukkebukta from the County Governor. No ethical permission is required from the Norwegian Animal Research Authority for collection with gill nets and the associated sacrifice of fish (FOR 1996-01-15 nr 23, the Norwegian Ministry of Agriculture and Food).

### Analysis of Gill Raker Number

For the present dataset, the number of gill rakers represents the only phenotypic trait that could be associated with individual genotypes. The number of gill rakers is an important trait involved in trophic utilization via the feeding efficiency [30], [32], [53], [54] and in coregonids, it has been shown to be heritable [55], [56] and influenced by diversifying selection [23], [57]. Moreover, since sympatric divergence in this system is promoted by ecological opportunity [54] and number of gill rakers contribute to the local adaptation [23], this trait could directly or indirectly impact on the extent of reproductive isolation. Thus, Kahilainen *et al* [54], demonstrated that increasing number of gill rakers facilitates the foraging of small prey, particularly zooplankton, and that fish with a high number of gill rakers generally are associated with a pelagic, zooplanktivore niche, whereas fish with lower gill raker numbers are associated with benthic habitat and diet utilization. Densely rakered gills are more likely to be clogged by bottom sediments, whereas sparsely rakered gills may not be able to filter small zooplankton efficiently [54]. In a field experiment demonstrating extrinsic post-zygotic isolation mechanism, three-spined stickleback hybrids showed slower growth rate as compare to the parental ones as a consequence of inheritance of intermediate phenotypic traits (including gill rakers) which likely resulted in lower fitness [19]. We used this trait to examine the potential breakdown of morphological differentiation by exploring the modality of the gill raker number distribution over the two sampling years (1993 and 2008). Kernel density estimation was used to produce smoothed gill raker distributions. A kernel smoother estimates the underlying probability density function for the given dataset and identifies the number of distributions. This was achieved in R using the data smoothing function density [58]. Secondly, we employed a model-based clustering method for R (MCLUST version 3) [59] to determine the number of morphological groups represented in the two sampling years. The data were fitted to models with one or a mixture of up to three Gaussian distributions. The MCLUST functions for univariate data consist of only two models with equal (denoted E) or varying variance (denoted V), respectively. The best model was selected based on the Bayesian Information Criterion (BIC). We calculated ΔBIC for each population by estimating the difference between BIC for the best and the next best model. Following Kass and Raftery [59], ΔBIC >10 was adopted as very strong support for the best model, 6< ΔBIC <10 as strong support, 2< ΔBIC <6 as moderate support, and ΔBIC <2 as equivalent support for the best and the next best model.

### Microsatellite Analysis

The total DNA was extracted from gill lamellae using E.Z.N.A E-Z 96 Tissue DNA Kit (Omega-Biotek) following the manufacturer’s protocol. Individuals were genotyped at 20 polymorphic microsatellite loci: BFRO-018 [60], BWF1, BWF2 [61], Cla-Tet01, Cla-Tet03, Cla-Tet06, Cla-Tet09, Cla-Tet10, Cla-Tet13, Cla-Tet15, Cla-Tet17, Cla-Tet18 [62], Cocl-Lav04, Cocl-Lav06, Cocl-Lav10, Cocl-Lav18, Cocl-Lav27, Cocl-Lav49, Cocl-Lav52 [63], C2-157 [64]. The loci were co-amplified in four 2.5 µl PCR multiplex reactions as described earlier [65] using the QIAGEN Multiplex PCR kit following the manufacturer’s protocol. The PCR products were denatured in Hi-Di Formamide, containing LIZ-500 internal size standard (Applied Biosystems) and separated using an ABI-3130×l Genetic Analyzer (Applied Biosystems). The alleles were scored using the automatic binning function, with predefined bins, as implemented in Gene-Mapper 3.7 software (Applied Biosystems). All alleles were subsequently verified visually by two independent persons. In addition, the included replicate and blank samples were manually verified to ensure the validity of the data. Finally, we manually verified all identified private alleles to ensure that they are not an artefact from inconsistent scoring.

### Statistical Analyses

Micro-Checker 2.2.3 [66], employing 1,000 bootstraps, was used to check for null alleles and genotyping errors. Loci showing null alleles were removed from the dataset before subsequent analysis.

Number of individual clusters (populations) at each of the two sampling years were inferred by STRUCTURE 2.2.3 [67]. We calculated the number of populations represented (K) and the individual admixture coefficients (q) of individuals belonging to the pure parental populations of respectively LSR, DR, or genetic hybrids of the two whitefish morphs. We used an admixture model with correlated allele frequency with burn-in period of 10,000 and number of iterations of 50,000 in five replicates. The statistical significance for the likely number of clusters in each of the two sampling years was tested by Kruskal-Wallis non-parametric test employed in SPSS [68] by comparing the ln Pr (*X*|*Κ*) values for each of 5 runs for variable K. We applied threshold values for q at 0.2 and 0.8 to categorise the individuals as hybrids or pure, with individuals having q-values between 0.2 and 0.8 being considered as hybrids and the others as pure individuals (q ≤0.2 and q ≥0.8). These threshold q-values have been shown by simulation studies to provide the most efficient detection of hybrids [69], as increasing the threshold q-value will misclassify the hybrids as pure individuals.

The populations defined from the q-values were used for further statistical analyses (see Table S1 and Table S2). Expected heterozygosity (H_e_) was estimated using GENEPOP 4.1 [70], [71]. The exact test [72] implemented in GENEPOP 4.1 was used to test for deviations from Hardy-Weinberg and linkage equilibrium within each population, and the corresponding P (probability value) was obtained by following Markov chain conditions as 10,000 dememorisation, 200 batches and 10,000 iterations. Estimates were corrected for multiple tests using sequential Bonferroni corrections as described by [73]. Allelic richness (A_R_) was estimated accounting for differences in sample sizes using the rarefaction procedure [74] as implemented in HP-RARE [75]. The estimates calculated here were based on the smallest number of samples (N = 27 in Sb93_Hybrids). The R function cor.test [58] was used to test the association between the gill raker numbers and admixture values (q-values).

## Results

### Analysis of Gill Raker Numbers

**Figure 2:**
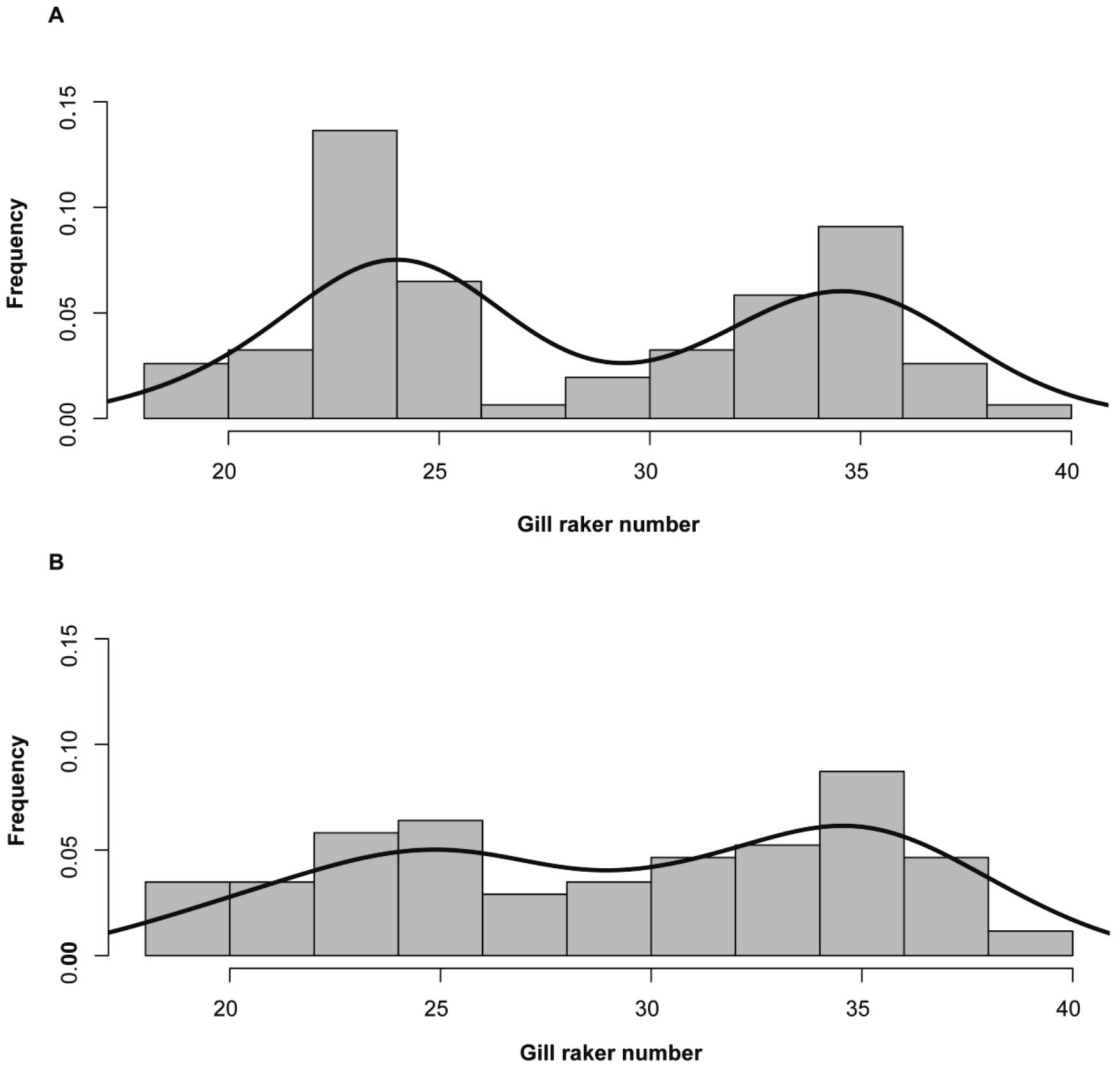
Frequency distribution of gill raker numbers in DR-LSR whitefish morph-pair samples collected in 1993 (A) and 2008 (B) from Lake Skrukkebukta. The solid line on the histogram indicates the kernel density function.

The kernel density plots of gill raker numbers over the 15 years revealed a bimodal distribution with modes growing closer with time since invasion (Figure 2A and 2B). The MCLUST analysis of frequencies of gill raker numbers between the LSR and DR whitefish followed a bimodal distribution before and after the vendace invasion (2008: ΔBIC = 9; 1993: ΔBIC = 8.4).

### Genetic Analysis

Four loci showed departures from Hardy-Weinberg equilibrium after Bonferroni corrections (Cla-Tet10, Cla-Tet17, Cla-Tet15 and Cocl-lav52), most likely due to null alleles or stuttering as indicated in the Micro-Checker analysis. These loci were, therefore, discarded from subsequent analyses. Summary statistics for the microsatellite loci and populations are listed in Table S1 and Table S2.

**Figure 3:**
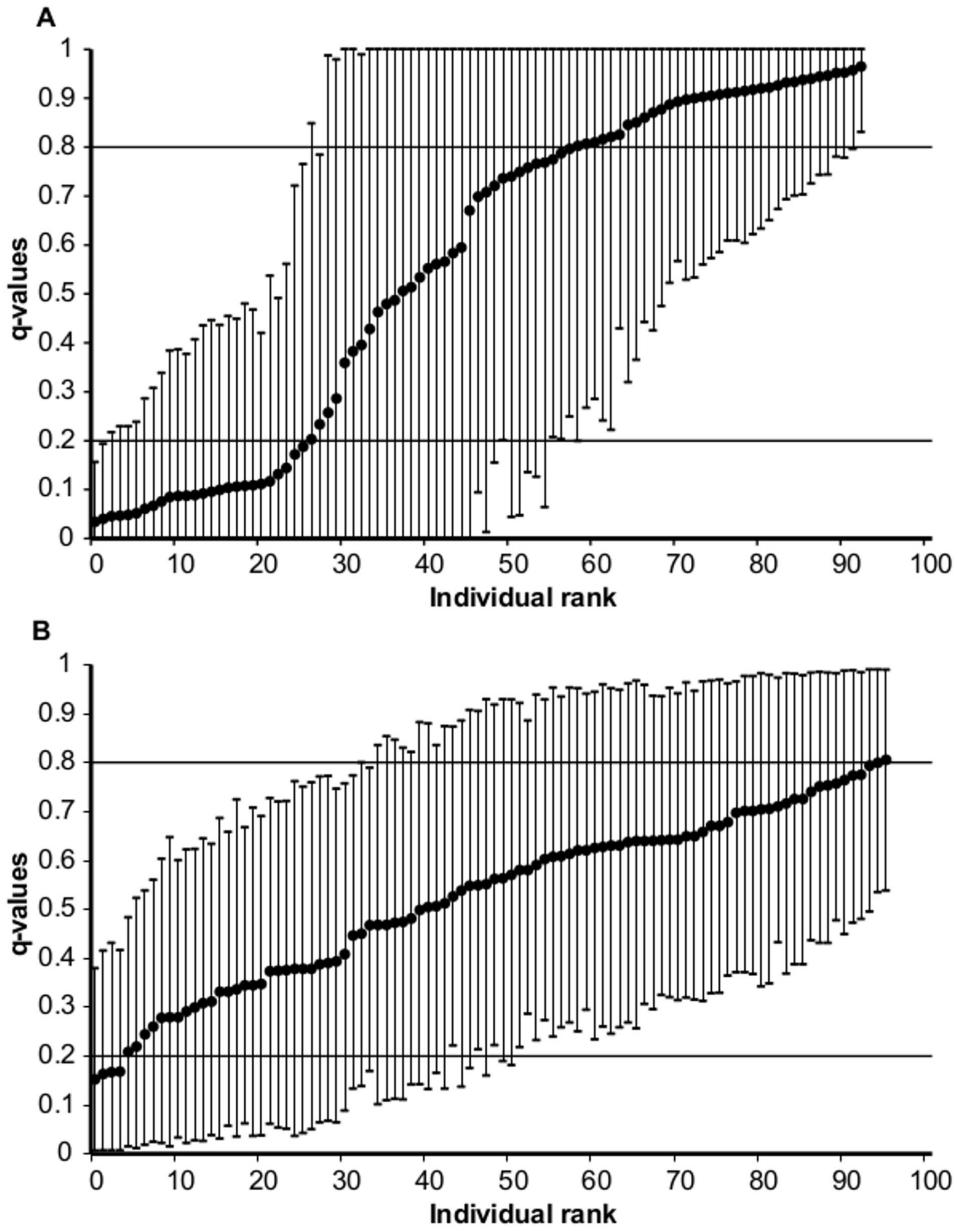
Frequency distribution of individual admixture proportions of DR-LSR whitefish morph-pair upon arrival (1993, A) and after (2008, B) a biological invasion. The q-values indicate admixture proportions with the associated 90% credible intervals as estimated by STRUCTURE 2.2.3 [67]. The solid horizontal lines indicate the threshold q-values for identifying hybrids and pure whitefish morphs.

The STRUCTURE analysis of temporal data confirmed the presence of two populations in the 1993 samples. Mean ln Pr (*X*|*Κ*) was significantly smaller for K = 2 (*mean* ln Pr (*X*|*Κ*) ± S.D.* = *−4467.58±9.91, Kruskal-Wallis test, P<0.05) as compared to either K = 1 (*mean* ln Pr (*X*|*Κ*) ± S.D.* = *−4505.46±0.76) or K = 3 *(mean* ln Pr (*X*|*Κ*) ± S.D.* = *−4567.96±57.94). However, in 2008, the most likely number of population was K = 1 *(mean* ln Pr (*X*|*Κ*) ± S.D.* = *−4706.78±0.55, Kruskal -Wallis test, P<0.05) rather than K = 2 (*mean* ln Pr (*X*|*Κ*) ± S.D. = −4824.6±19.28) or K = 3 (*mean* ln Pr (*X*|*Κ*) ± S.D.* = *−4812.42±17.18). The proportion of individuals assigned to one of the parental categories based on the threshold q-values (0.2/0.8) was 66% in 1993, mostly with non-overlapping 90% credible intervals (Figure 3A). In contrast, in 2008 all the individuals revealed q-values within the range from 0.2 to 0.8 with overlapping 90% credible intervals (Figure 3B).

**Figure 4:**
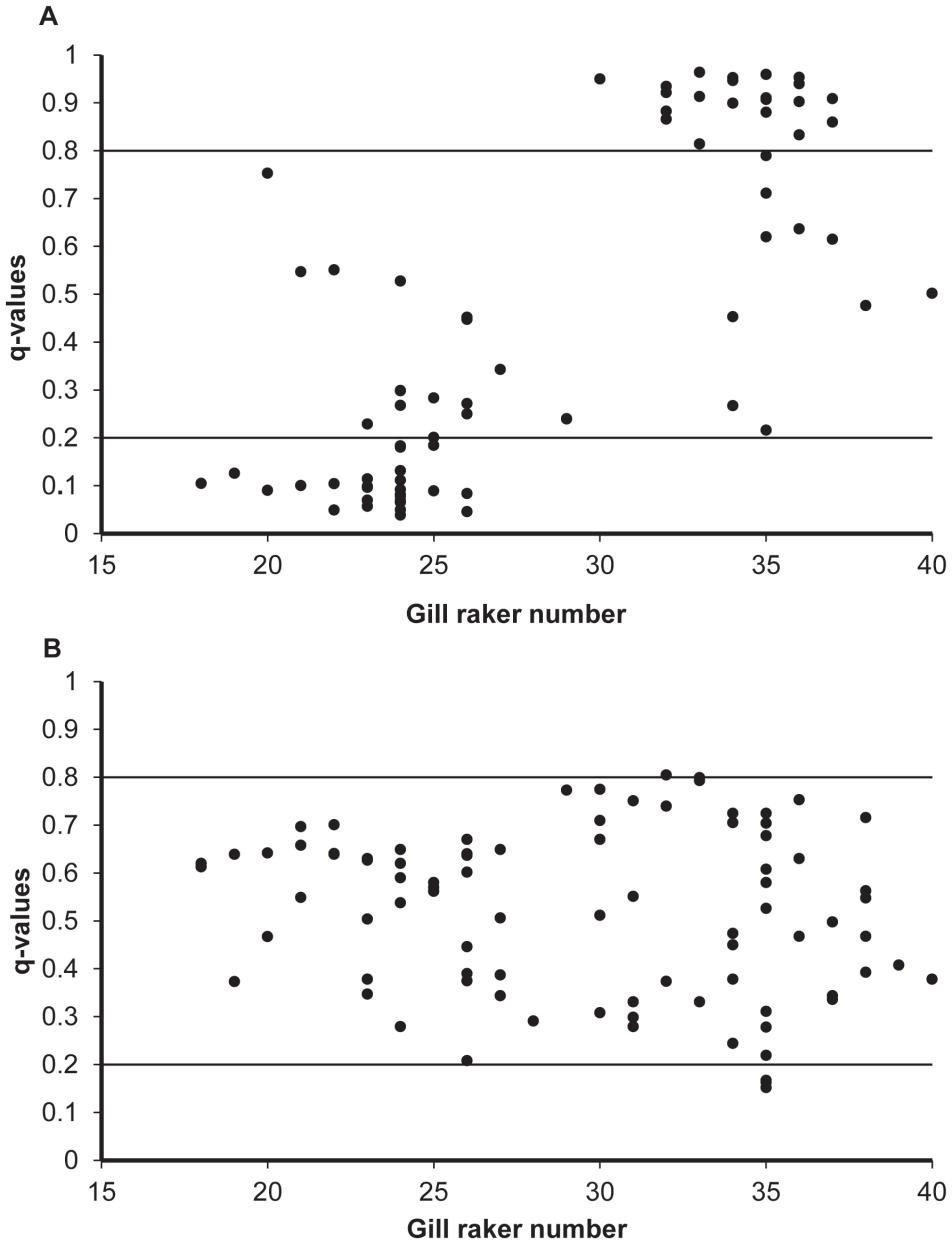
Scatter plots comparing genetic and morphological data of DR-LSR whitefish morph-pair at arrival (1993, A) and after (2008, B) a biological invasion. The individual admixture values (q-values) estimated by Structure 2.2.3 [67] are plotted against the gill raker numbers. The q-values indicate admixture proportions of individuals. The solid horizontal lines indicate the threshold q-values to identify hybrids and pure whitefish morphs.

The scatter plot of individual gill raker number and individual admixture coefficients (STRUCTURE q-values) showed two genetically and morphologically well differentiated groups of individuals in 1993 (Figure 4A). The individuals sampled in 2008, however, displayed no clear pattern of correlation between the individual admixture coefficients and gill raker number (Figure 4B). Support for this scenario was given by the correlation test for which a significant correlation between gill raker number and admixture values was observed in 1993 (Pearson correlation coefficient, r = 0.73, P<0.05), but not in 2008 (Pearson correlation coefficient, r = −0.177, P>0.05).

## Discussion

The results of this study suggest a breakdown of reproductive isolation between DR and LSR whitefish within a 15-year period following the invasion of a superior trophic competitor, vendace. Most noteworthy, a Bayesian admixture analysis of the genetic data failed to separate the DR and LSR whitefish in the 2008 sample, whereas a clear separation of the two populations was evident in 1993. Furthermore, population-pure individuals, i.e. genetically assigned as either LSR or DR whitefish, were nearly absent in 2008 compared to a frequency of approximately 66% pure individuals revealed in the 1993 sample. Also, the association between gill raker numbers and genotypic data was strong and significant in 1993, whereas a much weaker and non-significant correlation was observed in 2008. This reflects a breakdown of the association between the genotype and the phenotypic trait. The high frequency of hybrids between the LSR and DR whitefish in 2008 suggests that hybridization may have contributed to a decrease in the abundance of the DR whitefish observed after the vendace invasion [35].

Increased hybridization should presumably promote a collapse in the divergent pattern of gill raker numbers in the two morphs since hybrids would be expected to consist of intermediate phenotypes in respect to this heritable trait. In lakes harbouring reproductively isolated LSR and DR whitefish, the gill raker numbers generally follow a distinct bimodal distribution pattern [30], [32]. In the present study, the LSR and DR whitefish showed a bimodal distribution pattern of the gill rakers in both sampling years, even though all individuals in 2008 were assigned as hybrids according to the genetic analyses. A likely explanation for this apparent contradiction may be the few generations passed since the first vendace observation (i.e. approximately three generations over the 15 year time), not allowing enough time for intermediate phenotypes to accumulate (but see the discussion part on association between gill raker numbers and genotypes). Notwithstanding, in the 2008 sample the two modes in the gill-raker distribution representing DR and LSR whitefish, are less distinct and located closer to each other compared to the 1993 sample. This may suggest an on-going process towards a collapse in the bimodal gill-raker number distribution. An alternate, albeit non-exclusive, explanation could be selection against intermediate phenotypes as the gill raker number is a trophic trait influenced by natural disruptive selection ( [23], but see [76]). The gill raker number has a polygenic basis [77] and is expected to follow a complex pattern of inheritance and expression relative to other more discrete traits. The breakdown of the bimodal distribution pattern of gill raker numbers in hybridizing whitefish may, therefore, take longer than the rapid collapse in the divergent distribution of neutral microsatellites, as observed in the present study. This is because the homogenizing effect of hybridization may be delayed by the counteracting effect of disruptive natural selection. Such a mechanism was also suggested from a study involving two ecotypes of rainbow smelt (*Osmerus mordax*), in which natural selection maintained the adaptive phenotypic differences (including gill raker counts) in spite of the presence of high gene flow [78].

A genetic analysis (five microsatellite loci) combined with analysis of a phenotypic trait (body shape) spanning over a 25 year period, revealed a collapse of a limnetic-benthic species-pair of three-spined sticklebacks into a hybrid swarm [17]. Using STRUCTURE analysis as a proxy for departures from Hardy-Weinberg equilibrium (HWE) and linkage disequilibrium (LD), the study also showed a decline in linkage disequilibrium across years following the collapse. This decline was not evident in the pairwise loci comparisons nor within years. Likewise, the authors did not identify any decrease in observations of HWE after the collapse. In the present study involving 16 microsatellite loci, no deviations from HWE and presence of LD among loci were identified. However, the STRUCTURE output and scatterplot of q-values corroborate assumptions of a weakening of genetic differentiation between the whitefish morphs after the vendace invasion. Thus, this observation supports our expectation of loss of genetic differentiation between the LSR and DR whitefish.

The whitefish morphs are differentiated based on the number and morphology of the gill rakers as well as their morphological appearance [30], [37], [52]. Microsatellite studies have repeatedly reported genetic differentiation between DR and LSR whitefish in various lakes in northern Norway [21], [34]. Hence, there is reason to assume that hybridization and gene flow between the sympatric whitefish morphs should promote a collapse of the association between genetic and phenotypic traits (in our case gill raker numbers) due to increased admixture. In the analysis of the three-spined stickleback species-pairs from Enos Lake, the hybrid swarms showed no or little association between body morphology and genetic scores [17]. Herein, the lack of associations between gill raker numbers and q-values in 2008 agrees with the above findings and supports our expectation of a breakdown of the association between morphology and genotype frequencies in the whitefish.

Nonetheless, information about spawning behaviour and the nature of reproductive isolation mechanisms between the DR and LSR whitefish in northern Fennoscandian lakes remain speculative at this time. However, Table 1 summarizes putative reproductive isolation mechanisms between DR and LSR whitefish morphs based on available information from whitefishes and other postglacial fish species. Time and/or place of spawning are considered as an important prezygotic isolation mechanisms between whitefish morphs [7], [46], similarly as for other postglacial fishes like Arctic charr [22]. However, these mechanisms do not seem to be similar across different study lakes [46], [79]. Local lake conditions such as fluctuations in water temperature, bottom substrate and depth may be important factors in determining spawning time and place [46]. Svärdson [37] suggested that the differences in spawning time or place are important reproductive isolation mechansims in whitefish in Swedish lakes. A similar mechanism has been reported in Alpine whitefish species, which spawn at different depths ( [7] and Table 1). In Lake Stuorajavri (northern Norway), the LSR whitefish comprised 98% of the spawning whitefish caught at two different spawning grounds (R. Knudsen, unpublished), suggesting that the two morphs at least have separate spawning locations.

The fact that sympatric adults of DR and LSR whitefish differ in size suggests that size-assortative mating may be an important mechanism maintaining reproductive isolation in the face of gene flow (Table 1). Size assortative mating has been described as a pre-zygotic isolation mechanism between kokanee and sockeye salmon (*Oncorhynchus nerka*), as well as in other whitefish sympatric forms (*Coregonus* spp) [37], [48]. Microhabitat segregation of spawning site may be another possible mechansims of premating reproductive isolation as suggested by various studies [17], [45], [80].

Information regarding extrinsic and intrinsic post-zygotic reproductive isolation in coregonids exists from studies of North American lake whitefish [50], [51] and whitefish (*Coregonus* spp.) from the Swiss Alps [20]. Furthermore, severe genomic incompatibilities and related differential embryonic mortality have been reported in the hybrids of parents belonging to two glacial races of lake whitefish ( [50], [51], Table 1). However, no such intrinsic hybrid inviability was reported from a study of Alpine whitefish although the hybrids showed discrepancies in hatching time [20]. Hence, the authors suggested that hatching time could be a powerful selective force against hybrids in Alpine whitefish [20]. Another extrinsic post-zygotic reproductive isolation mechanism relates to ecological selection against hybrids or ecological inviability. Ecological inviability is imputable to reduced foraging efficiency due to the intermediate gill rakers or absence of appropriate ecological niches [19]. Considering the young evolutionary age (11700-5800 years before present) of the northern Fennoscandian whitefish monophyletic lineage [21], [33] and previous results in Alpine whitefish [20], it is plausible that the speciation process has not proceeded far enough to develop a strong or complete reproductive isolation between the sympatric whitefish morphs. The on-going or incomplete speciation process is a key signature of young northern post-glacial systems as compared to systems of an older evolutionary age. We propose, therefore, that our results suggest a breakdown of reproductive isolation between sympatric whitefish morph-pairs, further leading to a hybrid swarm triggered by the competitive interaction between native whitefish and non-native vendace.

The genetic architecture of the reproductive isolation mechanisms is largely unknown in European whitefish. In ecological speciation, the traits offering reproductive isolation must either be genetically correlated with traits under disruptive selection, or the traits offering the adaptive advantage should drive the reproductive isolation pleotropically. In such a situation, reproductive isolation may be achieved as a result of alleles that allow adaptation to a particular niche also will govern the mate choice [81]. Otherwise, the progress towards the evolution of reproductive isolation between resource specialists would require a build-up of non-random association between the set of genes optimal for each resource and the genes for assortative mating. In other words, reproductive isolation requires linkage disequilibrium between the genes affecting viability and those affecting assortative mating [81]. This suggests that the genes responsible for gill raker numbers in European whitefish must be in association with the genes responsible for intrinsic reproductive isolation. While this has not been investigated in European whitefish, a recent study on North American whitefish showed that covariation in expression of genes belonging to the same gene network was associated with phenotypic variation both in swimming behaviour and water depth preference and in number of gill rakers [82]. Since differential habitat preference (e.g. water depth) could promote reproductive isolation, and since both depth preference and number of gill rakers co-vary in association within the same gene network, these results on lake whitefish provide the first evidence that gill raker numbers could be involved in reproductive isolation between sympatric whitefish forms.

The observed hybridization and apparent re-admixture of DR and LSR whitefish following the vendace invasion in the Pasvik watercourse may represent an example of “speciation in reverse” [1], [4]. A few other examples of “speciation in reverse” in fishes have been described from natural systems inhabited by Alpine whitefish, three-spined sticklebacks, ciscoes and cichlids [4], [15], [17], [25,]. However, the reason for the collapse is not known in most of the reported cases and is only suggested in the collapse of morph-pairs in three-spined sticklebacks from Enos Lake [16], [17] and from species reversal in Alpine whitefish [15]. In the former case, the collapse of the species pair has been attributed to a coinciding establishment of American signal crayfish (*Pascifasticus lenisculus*) that may have altered the habitat use, mating behaviour and/or trophic distribution of sticklebacks in the lake. In the latter case, Vonlanthen *et al*. [15] suggested that anthropogenic induced eutrophication has partly destroyed the spawning grounds and caused relaxation of divergent selection thereby facilitated the collapse of native whitefish species-pairs in several Alpine lakes. Thus, there is reason to suggest that the relegation of DR whitefish into the habitat of LSR whitefish with a subsequent genetic homogenization may represents speciation reversal.

At the genomic level, the observed pattern of putative speciation reversal may be attributed to differential divergence at the local or global genomic scale, referred to as growth of genomic islands [83]. Genomic islands are genomic regions exhibiting greater differentiation than expected under neutrality. An ecological speciation model (with gene flow) of “genomic islands of divergence” predicts that the genes affecting the local adaptation and reproductive isolation may reside within the genomic clusters of islands [84]. Correlated response to selection particularly by virtue of linkage disequilibrium is most likely to reverse after the event of secondary contact. Recombination will break the larger genomic islands into smaller parts, but the genes under selection remains isolated in smaller linkage regions. In this scenario, the process may not be termed “speciation reversal” but would rather reflect the decay of genomic islands into smaller numbers and sizes. However, both scenarios are not necessarily exclusive as decay of genomic islands of divergence could actually be the key molecular mechanisms causing speciation reversal. This, however, remains to be investigated.

## Conclusions

Our study suggests an apparent breakdown of reproductive isolation between two sympatric European whitefish morphs over a relatively short time span of 15 years (i.e. within three generations) following the invasion of a superior resource competitor (vendace) in a subarctic lake. Further studies are necessary to understand the mechanisms and dynamics that are weakening the reproductive isolation and the potential evolutionary consequences for the whitefish populations in the lakes of the Pasvik watercourse. In addition, a genome wide study would be necessary to shed more light on the effect and dynamics of admixture at the genomic level.

## Supporting Information

Table S1 Summary statistics for each locus and four populations from 1993 and 2008.
(DOCX)

Table S2 Details of population and basic genetic diversity measures in four populations from 1993 and 2008.
(DOCX)

## References

[1] Coyne JA, Orr HA (2004) Speciation. Sunderland, Massachusetts: Sinauer Associate Inc.

[2] Schluter D (2001) Ecology and the origin of species. Trends Ecol Evol 16: 372–380. 11403870 10.1016/s0169-5347(01)02198-x

[3] Rundle HD, Nosil P (2005) Ecological speciation. Ecol Lett 8: 336–352.

[4] Seehausen O (2006) Conservation: Losing biodiversity by reverse speciation. Curr Biol 16: R334–R337. 16682344 10.1016/j.cub.2006.03.080

[5] Wimberger PH (1994) Trophic polymorphisms, plasticity and speciation in vertebrates. In: Fresh KL, Fresh RJ, editors. Theory and application in fish feeding ecology. Colombia: University of South Carolina Press. 19–43.

[6] Ritchie MG (2007) Sexual selection and speciation. Annu Rev Ecol Syst 38: 79–102.

[7] Vonlanthen P, Roy D, Hudson AG, Largiader CR, Bittner D, et al (2009) Divergence along a steep ecological gradient in lake whitefish (*Coregonus sp*.). J Evol Biol 22: 498–514. 19170819 10.1111/j.1420-9101.2008.01670.x

[8] Seehausen O, Mayhew PJ, Van Alphen JJM (1999) Evolution of colour patterns in East African cichlid fish. J Evol Biol 12: 514–534.

[9] Coyne JA, Crittenden AP, Mah K (1994) Genetics of a pheromonal difference contributing to reproductive isolation in Drosophila. Science 265: 1461–1464. 8073292 10.1126/science.8073292

[10] Boughman JW (2001) Divergent sexual selection enhances reproductive isolation in sticklebacks. Nature 411: 944–948. 11418857 10.1038/35082064

[11] Seehausen O, vanAlphen JJM, Witte F (1997) Cichlid fish diversity threatened by eutrophication that curbs sexual selection. Science 277: 1808–1811.

[12] Gilman RT, Behm JE (2011) Hybridization, species collpase, and species reemergence after disturbance to premating mechanisms of reproductive isolation. Evolution 65: 2592–2605. 21884058 10.1111/j.1558-5646.2011.01320.x

[13] Seehausen O, Takimoto G, Roy D, Jokela J (2008) Speciation reversal and biodiversity dynamics with hybridization in changing environments. Mol Ecol 17: 30–44. 18034800 10.1111/j.1365-294X.2007.03529.x

[14] Vines TH, Schluter D (2006) Strong assortative mating between allopatric sticklebacks as a by-product of adaptation to different environments. Philos Trans R Soc Lond B Biol Sci 273: 911–916. 10.1098/rspb.2005.3387 PMC1560240 16627275

[15] Vonlanthen P, Bittner D, Hudson AG, Young KA, Muller R, et al (2012) Eutrophication causes speciation reversal in whitefish adaptive radiations. Nature 482: 357–362. 22337055 10.1038/nature10824

[16] Velema GJ, Rosenfeld JS, Taylor EB (2012) Effects of invasive American signal crayfish (*Pacifastacus leniusculus*) on the reproductive behaviour of threespine stickleback (*Gasterosteus aculeatus*) sympatric species pairs. Can J Zool 90: 1328–1338.

[17] Taylor EB, Boughman JW, Groenenboom M, Sniatynski M, Schluter D, et al (2006) Speciation in reverse: morphological and genetic evidence of the collapse of a three-spined stickleback (*Gasterosteus aculeatus*) species pair. Mol Ecol 15: 343–355. 16448405 10.1111/j.1365-294X.2005.02794.x

[18] Bernatchez L, Renaut S, Whiteley AR, Derome N, Jeukens J, et al (2010) On the origin of species: insights from the ecological genomics of lake whitefish. Philos Trans R Soc Lond B Biol Sci 365: 1783–1800. 20439281 10.1098/rstb.2009.0274 PMC2871888

[19] Hatfield T, Schluter D (1999) Ecological speciation in sticklebacks: environment-dependent hybrid fitness. Evolution 53: 866–873. 28565618 10.1111/j.1558-5646.1999.tb05380.x

[20] Woods PJ, Muller R, Seehausen O (2009) Intergenomic epistasis causes asynchronous hatch times in whitefish hybrids, but only when parental ecotypes differ. J Evol Biol 22: 2305–2319. 19824932 10.1111/j.1420-9101.2009.01846.x

[21] Østbye K, Amundsen P-A, Bernatchez L, Klemetsen A, Knudsen R, et al (2006) Parallel evolution of ecomorphological traits in the European whitefish *Coregonus lavaretus* (L.) species complex during postglacial times. Mol Ecol 15: 3983–4001. 17054498 10.1111/j.1365-294X.2006.03062.x

[22] Klemetsen A (2010) The charr problem revisited: exceptional phenotypic plasticity promotes ecological speciation in postglacial lakes. Freshw Rev 3: 49–74.

[23] Bernatchez L (2004) Ecological theory of adaptive radiation: an empirical assessment from coregonine fishes (Salmoniformes). In: Hendry AP, Stearns SC, editors. Evolution illuminated, salmon and their relatives. New York: Oxford University Press. 175–207.

[24] Behm JE, Ives AR, Boughman JW (2010) Breakdown in postmating isolation and the collapse of a species pair through hybridization. Am Nat 175: 11–26. 19916869 10.1086/648559

[25] Todd TN, Stedman RM (1989) Hybridization of ciscoes (*Coregonus* spp) in Lake Huron. Can J Zool 67: 1679–1685.

[26] Grant PR, Grant BR (1996) Speciation and hybridization in island birds. Philos Trans R Soc Lond B Biol Sci 351: 765–772.

[27] Rhymer JM, Simberloff D (1996) Extinction by hybridization and introgression. Annu Rev Ecol Syst 27: 83–109.

[28] Senn HV, Barton NH, Goodman SJ, Swanson GM, Abernethy KA, et al (2010) Investigating temporal changes in hybridization and introgression in a predominantly bimodal hybridizing population of invasive sika (*Cervus nippon*) and native red deer (*C.elaphus*) on the Kintyre Peninsula, Scotland. Mol Ecol 19: 910–924. 20102517 10.1111/j.1365-294X.2009.04497.x

[29] Grant PR (1993) Hybridization of Darwin finches on Isla-Daphne Major, Galapagos. Philos Trans R Soc Lond B Biol Sci 340: 127–139. 10.1098/rstb.2014.0287 PMC4360115 25750230

[30] Amundsen P-A, Bøhn T, Vaga GH (2004) Gill raker morphology and feeding ecology of two sympatric morphs of European whitefish (*Coregonus lavaretus*). Ann Zool Fennici 41: 291–300.

[31] Kahilainen K, Østbye K (2006) Morphological differentiation and resource polymorphism in three sympatric whitefish *Coregonus lavaretus* (L.) forms in a subarctic lake. J Fish Biol 68: 63–79.

[32] Siwertsson A, Knudsen R, Kahilainen KK, Praebel K, Primicerio R, et al (2010) Sympatric diversification as influenced by ecological opportunity and historical contingency in a young species lineage of whitefish. Evol Ecol Res 12: 929–947.

[33] Østbye K, Bernatchez L, Naesje TF, Himberg KJM, Hindar K (2005) Evolutionary history of the European whitefish *Coregonus lavaretus* (L.) species complex as inferred from mtDNA phylogeography and gill-raker numbers. Mol Ecol 14: 4371–4387. 16313599 10.1111/j.1365-294X.2005.02737.x

[34] Præbel K, Knudsen R, Siwertsson A, Karhunen M, Kahilainen KK, et al (2013) Ecological speciation in postglacial European whitefish: rapid adaptive radiations into the littoral, pelagic, and profundal lake habitats. Ecol Evol 3: 4970–4986. 24455129 10.1002/ece3.867 PMC3892361

[35] Bøhn T, Amundsen P-A, Sparrow A (2008) Competitive exclusion after invasion? Biol Invasions 10: 359–368.

[36] Bøhn T, Sandlund OT, Amundsen P-A, Primicerio R (2004) Rapidly changing life history during invasion. Oikos 106: 138–150.

[37] Svärdson G (1979) Speciation of Scandinavian Coregonus. Report of the Institute of Freshwater Research, Drottningholm 57: 3–95.

[38] Amundsen P-A, Staldvik FJ, Reshetnikov YS, Kashulin N, Lukin A, et al (1999) Invasion of vendace *Coregonus albula* in a subarctic watercourse. Biol Conserv 88: 405–413.

[39] Mutenia A, Salonen E (1992) The vendace (*Coregonus albula* L.), a new species in the fish community and fisheries of Lake Inari. Pol Arch Hydrobiol 39: 797–805.

[40] Præbel K, Gjelland KØ, Salonen E, Amundsen P-A (2013) Invasion genetics of vendace (*Coregonus albula* (L.)) in the Inari-Pasvik watercourse: revealing the origin and expansion pattern of a rapid colonization event. Ecol Evol 3: 1400–1412. 23762524 10.1002/ece3.552 PMC3678492

[41] Gjelland KØ, Bøhn T, Amundsen PA (2007) Is coexistence mediated by microhabitat segregation? An in-depth exploration of a fish invasion. J Fish Biol 71: 196–209.

[42] Amundsen P-A, Siwertsson A, Primicerio R, Bøhn T (2009) Long-term responses of zooplankton to invasion by a planktivorous fish in a subarctic watercourse. Freshwater Biol 54: 24–34.

[43] Bøhn T, Amundsen P-A (1998) Effects of invading vendace (*Coregonus albula* L.) on species composition and body size in two zooplankton communities of the Pasvik river system, northern Norway. J Plankton Res 20: 243–256.

[44] Varnavskaya NV, Wood CC, Everett RJ, Wilmot RL, Varnavsky VS, et al (1994) Genetic differentiation of subpopulations of sockeye salmon (*Oncorhynchus nerka*) within lakes of Alaska, British-Columbia, and Kamchatka, Russia. Can J Fish Aquat Sci 51: 147–157.

[45] Zimmerman CE, Reeves GH (2000) Population structure of sympatric anadromous and nonanadromous *Oncorhynchus mykiss*: evidence from spawning surveys and otolith microchemistry. Can J Fish Aquat Sci 57: 2152–2162.

[46] Svärdson G (1965) The coregonid problem. VII. The isolating mechanisms in sympatric species. Report of the Institute of Freshwater Research, Drottningholm 46: 95–123.

[47] McKinnon JS, Mori S, Blackman BK, David L, Kingsley DM, et al (2004) Evidence for ecology’s role in speciation. Nature 429: 294–298. 15152252 10.1038/nature02556

[48] Foote CJ (1988) Male mate choice dependent on male size in salmon. Behaviour 106: 63–80.

[49] Abbott R, Albach D, Ansell S, Arntzen JW, Baird SJE, et al (2013) Hybridization and speciation. J Evol Biol 26: 229–246. 23323997 10.1111/j.1420-9101.2012.02599.x

[50] Lu GQ, Bernatchez L (1998) Experimental evidence for reduced hybrid viability between dwarf and normal ecotypes of lake whitefish (*Coregonus clupeaformis* Mitchill). Proc R Soc Lond B Biol Sci 265: 1025–1030.

[51] Rogers SM, Bernatchez L (2006) The genetic basis of intrinsic and extrinsic post-zygotic reproductive isolation jointly promoting speciation in the lake whitefish species complex (*Coregonus clupeaformis*). J Evol Biol 19: 1979–1994. 17040396 10.1111/j.1420-9101.2006.01150.x

[52] Siwertsson A, Knudsen R, Amundsen PA (2012) Temporal stability in gill raker numbers of subarctic European whitefish populations. Advanc Limnol 63: 229–240.

[53] Harrod C, Mallela J, Kahilainen KK (2010) Phenotype-environment correlations in a putative whitefish adaptive radiation. J Anim Ecol 79: 1057–1068. 20487087 10.1111/j.1365-2656.2010.01702.x

[54] Kahilainen KK, Siwertsson A, Gjelland KØ, Knudsen R, Bøhn T, et al (2011) The role of gill raker number variability in adaptive radiation of coregonid fish. Evol Ecol 25: 573–588.

[55] Rogers SM, Bernatchez L (2007) The genetic architecture of ecological speciation and the association with signatures of selection in natural lake whitefish (*Coregonus sp* Salmonidae) species pairs. Mol Biol Evol 24: 1423–1438. 17404398 10.1093/molbev/msm066

[56] Svärdson G (1970) Significance of introgression in coregonid evolution. In: Lindsey CC, Woods CS, editors. Biology of Coregonid Fishes. Winnipeg: University of Manitoba Press. 33–59.

[57] Østbye K, Næsje TF, Bernatchez L, Sandlund OT, Hindar K (2005) Morphological divergence and origin of sympatric populations of European whitefish (*Coregonus lavaretus* L.) in Lake Femund, Norway. J Evol Biol 18: 683–702. 15842498 10.1111/j.1420-9101.2004.00844.x

[58] R Development Core Team (2012) R: A language and environment for statistical computing.

[59] Kass RE, Raftery AE (1995) Bayes Factors. J Am Stat Assoc 90: 773–795.

[60] Susnik S, Snoj A, Dovc P (1999) Microsatellites in grayling (*Thymallus thymallus*): comparison of two geographically remote populations from the Danubian and Adriatic river basin in Slovenia. Mol Ecol 8: 1756–1758. 10583841 10.1046/j.1365-294x.1999.00723-2.x

[61] Patton JC, Gallaway BJ, Fechhelm RG, Cronin MA (1997) Genetic variation of microsatellite and mitochondrial DNA markers in broad whitefish (*Coregonus nasus*) in the Colville and Sagavanirktok rivers in northern Alaska. Can J Fish Aquat Sci 54: 1548–1556.

[62] Winkler KA, Weiss S (2008) Eighteen new tetranucleotide microsatellite DNA markers for *Coregonus lavaretus* cloned from an alpine lake population. Mol Ecol Resour 8: 1055–1058. 21585970 10.1111/j.1755-0998.2008.02153.x

[63] Rogers SM, Marchand MH, Bernatchez L (2004) Isolation, characterization and cross-salmonid amplification of 31 microsatellite loci in the lake whitefish (*Coregonus clupeaformis*, Mitchill). Mol Ecol Notes 4: 89–92.

[64] Turgeon J, Estoup A, Bernatchez L (1999) Species flock in the North American Great Lakes: Molecular ecology of Lake Nipigon ciscoes (Teleostei : Coregonidae : *Coregonus*). Evolution 53: 1857–1871. 28565465 10.1111/j.1558-5646.1999.tb04568.x

[65] Præbel K, Westgaard J-I, Amundsen P-A, Siwertsson A, Knudsen R, et al (2013) A diagnostic tool for efficient analysis of population structure, hybridization and conservation status of European whitefish (*Coregonus lavaretus* (L.)) and vendace (*C. albula* (L.)). Fund Appl Limnol 64: 247–255.

[66] Van Oosterhout C, Hutchinson WF, Wills DPM, Shipley P (2004) MICRO-CHECKER: software for identifying and correcting genotyping errors in microsatellite data. Mol Ecol Resour 4: 535–538.

[67] Pritchard JK, Stephens M, Donnelly P (2000) Inference of population structure using multilocus genotype data. Genetics 155: 945–959. 10835412 10.1093/genetics/155.2.945 PMC1461096

[68] IBM Corp (2010) IBM SPSS Statistics for Windows, Version 19.0. Armonk, NY: IBM Corp.

[69] Vähä JP, Primmer CR (2006) Efficiency of model-based Bayesian methods for detecting hybrid individuals under different hybridization scenarios and with different numbers of loci. Mol Ecol 15: 63–72. 16367830 10.1111/j.1365-294X.2005.02773.x

[70] Raymond M, Rousset F (1995) Genepop (Version-1.2) Population genetics software for exact tests and ecumenicism. J Hered 86: 248–249.

[71] Rousset F (2008) GENEPOP ′ 007: a complete re-implementation of the GENEPOP software for Windows and Linux. Mol Ecol Notes 8: 103–106. 10.1111/j.1471-8286.2007.01931.x 21585727

[72] Guo SW, Thompson EA (1992) Performing the exact test of Hardy-Weinberg proportion for multiple alleles. Biometrics 48: 361–372. 1637966

[73] Rice WR (1989) Analyzing tables of statisitcal tests. Evolution 43: 223–225. 28568501 10.1111/j.1558-5646.1989.tb04220.x

[74] Hurlbert SH (1971) The non concept of species diversity: a critique and alternative parameters. Ecology 52: 577–586. 28973811 10.2307/1934145

[75] Kalinowski ST (2005) HP-RARE 1.0: a computer program for performing rarefaction on measures of allelic richness. Mol Ecol Notes 5: 187–189.

[76] Crowder LB (1984) Character displacement and habitat shift in a native cisco in southeastern Lake-Michigan - evidence for competition. Copeia: 878–883.

[77] Hatfield T (1997) Genetic divergence in adaptive characters between sympatric species of stickleback. Am Nat 149: 1009–1029. 18811261 10.1086/286036

[78] Saint-Laurent R, Legault M, Bernatchez L (2003) Divergent selection maintains adaptive differentiation despite high gene flow between sympatric rainbow smelt ecotypes (*Osmerus mordax* Mitchill). Mol Ecol 12: 315–330. 12535084 10.1046/j.1365-294x.2003.01735.x

[79] Bernatchez L, Vuorinen JA, Bodaly RA, Dodson JJ (1996) Genetic evidence for reproductive isolation and multiple origins of sympatric trophic ecotypes of whitefish (*Coregonus*). Evolution 50: 624–635. 28568931 10.1111/j.1558-5646.1996.tb03873.x

[80] Chouinard A, Pigeon D, Bernatchez L (1996) Lack of specialisation in trophic morphology between genetically differentiated dwarf and normal forms of lake whitefish (*Coregonus clupeaformis* Mitchill) in Lac de l′Est, Quebec. Can J Zool 74: 1989–1998.

[81] Rice WR (1984) Disruptive selection on habitat preference and the evolution of reproductive isolation: a simulation study. Evolution 38: 1251–1260. 28563785 10.1111/j.1558-5646.1984.tb05647.x

[82] Filteau M, Pavey SA, St-Cyr J, Bernatchez L (2013) Gene co-expression networks reveal key drivers of phenotypic divergence in lake whitefish. Mol Biol Evol 30: 1384–1396. 23519315 10.1093/molbev/mst053

[83] Gagnaire PA, Pavey SA, Normandeau E, Bernatchez L (2013) The genetic architecture of reproductive isolation during speciation-with-gene-flow in lake whitefish species pairs assessed by RAD sequencing. Evolution 67: 2483–2497. 24033162 10.1111/evo.12075

[84] Nosil P, Funk DJ, Ortiz-Barrientos D (2009) Divergent selection and heterogeneous genomic divergence. Mol Ecol 18: 375–402. 19143936 10.1111/j.1365-294X.2008.03946.x

