# Supplementary material for: Speciation Reversal in European Whitefish (Coregonus lavaretus (L.)) Caused by Competitor Invasion
Source: PLoS One. 2014 Mar 13;9(3):e91208. doi: 10.1371/journal.pone.0091208 (PMC3953381; doi:10.1371/journal.pone.0091208)
Supplement: Table S1 — Summary statistics for each locus and four populations from 1993 and 2008. (DOCX) [file pone.0091208.s001.docx]

**Supplementary Table**

**Table S1.** Summary of sample sizes (N), number of alleles (N_A_), observed (H_o_) and expected (H_e_) heterozygosity, and F_IS_-estimates of the whitefish populations at 16 microsatellite loci. Population (Pop): whitefish morphs (LSR: large sparsely rakered, DR: densely rakered, pure individuals (q<0.2 and q>0.8) and hybrids (q between 0.2-0.8)).

| **Pop** |  | **BWF1** | **BWF2** | **Cla-Tet03** | **Cla-Tet13** | **Cla-Tet18** | **Cocl-lav04** | **Cocl-lav06** | **Cocl-lav10** | **Cocl-lav27** | **BFRO-018** | **Cocl-lav18** | **Cocl-lav49** | **Cla-Tet06** | **Cla-Tet09** | **C2-157** | **Cla-Tet01** |
| --- | --- | --- | --- | --- | --- | --- | --- | --- | --- | --- | --- | --- | --- | --- | --- | --- | --- |
| **Sb93DR_Pure** | N | 28 | 28 | 28 | 28 | 28 | 28 | 28 | 28 | 28 | 28 | 28 | 28 | 28 | 28 | 28 | 28 |
|  | N_A_ | 5 | 6 | 6 | 6 | 4 | 3 | 5 | 3 | 3 | 5 | 2 | 6 | 10 | 10 | 5 | 10 |
|  | H_o_ | 0.929 | 0.643 | 0.786 | 0.643 | 0.393 | 0.250 | 0.750 | 0.536 | 0.321 | 0.571 | 0.357 | 0.750 | 0.929 | 0.857 | 0.679 | 0.750 |
|  | H_e_ | 0.726 | 0.674 | 0.739 | 0.640 | 0.355 | 0.223 | 0.720 | 0.559 | 0.275 | 0.608 | 0.375 | 0.652 | 0.767 | 0.855 | 0.778 | 0.844 |
|  | F_IS_ | -0.278 | 0.046 | -0.064 | -0.005 | -0.106 | -0.123 | -0.042 | 0.041 | -0.169 | 0.060 | 0.048 | -0.150 | -0.210 | -0.003 | 0.128 | 0.111 |
|  |  |  |  |  |  |  |  |  |  |  |  |  |  |  |  |  |  |
| **Sb93LSR_ Pure** | N | 33 | 33 | 33 | 33 | 33 | 33 | 33 | 33 | 33 | 33 | 33 | 33 | 33 | 33 | 33 | 33 |
|  | N_A_ | 7 | 8 | 10 | 8 | 8 | 5 | 7 | 3 | 5 | 5 | 2 | 7 | 17 | 11 | 11 | 12 |
|  | H_o_ | 0.697 | 0.758 | 0.788 | 0.848 | 0.485 | 0.121 | 0.455 | 0.424 | 0.364 | 0.485 | 0.212 | 0.788 | 0.939 | 0.818 | 0.818 | 0.909 |
|  | H_e_ | 0.727 | 0.726 | 0.808 | 0.761 | 0.474 | 0.117 | 0.618 | 0.555 | 0.338 | 0.563 | 0.190 | 0.773 | 0.888 | 0.846 | 0.793 | 0.902 |
|  | F_IS_ | 0.042 | -0.043 | 0.024 | -0.115 | -0.022 | -0.039 | 0.264 | 0.235 | -0.076 | 0.139 | -0.119 | -0.019 | -0.058 | 0.033 | -0.031 | -0.008 |
|  |  |  |  |  |  |  |  |  |  |  |  |  |  |  |  |  |  |
| **Sb93_Hybrids** | N | 32 | 32 | 32 | 32 | 32 | 32 | 32 | 32 | 32 | 32 | 32 | 32 | 32 | 32 | 32 | 32 |
|  | N_A_ | 8 | 9 | 10 | 7 | 6 | 4 | 7 | 3 | 3 | 6 | 2 | 8 | 20 | 12 | 8 | 13 |
|  | H_o_ | 0.750 | 0.813 | 0.844 | 0.750 | 0.156 | 0.250 | 0.500 | 0.594 | 0.156 | 0.469 | 0.313 | 0.781 | 0.906 | 0.781 | 0.906 | 0.844 |
|  | H_e_ | 0.799 | 0.770 | 0.845 | 0.755 | 0.149 | 0.277 | 0.597 | 0.590 | 0.147 | 0.463 | 0.375 | 0.748 | 0.913 | 0.795 | 0.818 | 0.882 |
|  | F_IS_ | 0.062 | -0.056 | 0.001 | 0.006 | -0.049 | 0.097 | 0.162 | -0.006 | -0.063 | -0.012 | 0.167 | -0.045 | 0.007 | 0.018 | -0.107 | 0.043 |
|  |  |  |  |  |  |  |  |  |  |  |  |  |  |  |  |  |  |
| **Sb08_Hybrids** | N | 95 | 96 | 96 | 96 | 96 | 96 | 94 | 96 | 96 | 96 | 96 | 96 | 96 | 96 | 96 | 95 |
|  | N_A_ | 8 | 8 | 14 | 11 | 10 | 5 | 8 | 4 | 6 | 5 | 3 | 9 | 23 | 13 | 13 | 17 |
|  | H_o_ | 0.659 | 0.761 | 0.696 | 0.761 | 0.359 | 0.163 | 0.611 | 0.554 | 0.272 | 0.598 | 0.380 | 0.813 | 0.824 | 0.837 | 0.793 | 0.846 |
|  | H_e_ | 0.787 | 0.757 | 0.792 | 0.795 | 0.418 | 0.180 | 0.751 | 0.598 | 0.263 | 0.580 | 0.383 | 0.759 | 0.889 | 0.821 | 0.814 | 0.908 |
|  | F_IS_ | 0.162 | -0.005 | 0.122 | 0.043 | 0.142 | 0.095 | 0.186 | 0.072 | -0.032 | -0.030 | 0.007 | -0.071 | 0.073 | -0.019 | 0.025 | 0.068 |
