# Supplementary material for: Speciation Reversal in European Whitefish (Coregonus lavaretus (L.)) Caused by Competitor Invasion
Source: PLoS One. 2014 Mar 13;9(3):e91208. doi: 10.1371/journal.pone.0091208 (PMC3953381; doi:10.1371/journal.pone.0091208)
Supplement: Table S2 — Details of population and basic genetic diversity measures in four populations from 1993 and 2008. (DOCX) [file pone.0091208.s002.docx]

**Supplementary Table**

**Table S2.** Details of population and basic genetic diversity measures in four populations from 1993 and 2008. Population: whitefish morphs (LSR: large sparsely rakered, DR: densely rakered, Pure individuals (q<0.2 and q>0.8) and hybrids (q between 0.2-0.8)), year of sampling (Year), sample size (N), observed heterozygosity (H_o_), expected heterozygosity (H_e_), rarefacted allelic richness (A_R_) , *p*-values of exact tests for deviations from expected Hardy-Weinberg proportions (H.-W.) of the studied populations.

| Population | Year | N | H_o_ | H_e_ | A_R_ | H-W test |
| --- | --- | --- | --- | --- | --- | --- |
| Sb93DR_Pure | 1993 | 28 | 0.634 | 0.623 | 5.56 | 0.443 |
| Sb93LSR_Pure | 1993 | 33 | 0.619 | 0.640 | 7.52 | 0.104 |
| Sb93_Hybrids | 1993 | 32 | 0.613 | 0.630 | 7.55 | 0.525 |
| Sb08_Hybrids | 2008 | 96 | 0.630 | 0.656 | 7.55 | 0.123 |
